# Rab26 controls secretory granule maturation and breakdown in *Drosophila*

**DOI:** 10.1007/s00018-022-04674-8

**Published:** 2023-01-04

**Authors:** Attila Boda, Luca Petra Varga, Anikó Nagy, Győző Szenci, Tamás Csizmadia, Péter Lőrincz, Gábor Juhász

**Affiliations:** 1 Department of Anatomy, Cell and Developmental Biology, Eötvös Loránd University, Budapest, Hungary; 2 Institute of Genetics, Biological Research Centre, Szeged, Hungary

**Keywords:** Rab26, Secretory granule, Crinophagy, Salivary gland, Mon1

## Abstract

At the onset of *Drosophila* metamorphosis, plenty of secretory glue granules are released from salivary gland cells and the glue is deposited on the ventral side of the forming (pre)pupa to attach it to a dry surface. Prior to this, a poorly understood maturation process takes place during which secretory granules gradually grow via homotypic fusions, and their contents are reorganized. Here we show that the small GTPase Rab26 localizes to immature (smaller, non-acidic) glue granules and its presence prevents vesicle acidification. Rab26 mutation accelerates the maturation, acidification and release of these secretory vesicles as well as the lysosomal breakdown (crinophagy) of residual, non-released glue granules. Strikingly, loss of Mon1, an activator of the late endosomal and lysosomal fusion factor Rab7, results in Rab26 remaining associated even with the large glue granules and a concomitant defect in glue release, similar to the effects of Rab26 overexpression. Our data thus identify Rab26 as a key regulator of secretory vesicle maturation that promotes early steps (vesicle growth) and inhibits later steps (lysosomal transport, acidification, content reorganization, release, and breakdown), which is counteracted by Mon1.

**Supplementary Information:**

The online version contains supplementary material available at 10.1007/s00018-022-04674-8.

## Introduction

In salivary gland cells of third-instar (L3) larvae of the fruit fly (*Drosophila melanogaster*), bulk production of secretory granules takes place that undergo a maturation process. These are secreted during puparium formation, and their content including “glue” proteins attaches the metamorphosing animal onto a solid surface. These proteins are encoded by the Sgs (Salivary gland secretion) genes, some of which are heavily glycosylated [1]. Sgs gene expression starts midway through the L3 stage in a 20-hydroxyecdysone (20E)-dependent manner [2]. Secretory granules originate from the Golgi apparatus and their size gradually increases, while their number decreases before being secreted [3, 4]. This maturation process is also 20E-dependent and reduces the amount of the glue granules from more than 10,000 to approximately 3000 per cell [5]. Their exocytosis is triggered by another peak of 20E and it requires calcium ions [6]. After bulk secretion is finished, a subset of large granules remains in the cytosol of prepupal salivary gland cells. These are non-secreted granules committed to lysosomal degradation via crinophagy [4].

The details of secretory granule maturation are still incompletely understood. Previously clathrin and clathrin adaptor protein complex 1 (AP-1) were found to be required for secretory granule biogenesis, while loss of type II phosphatidylinositol 4-kinase (PI4KII) resulted in immature mucin-containing glue granules [3, 7]. It was recently demonstrated that many proteins functioning in the endolysosomal pathway are required for secretory granule maturation in the *Drosophila* salivary gland [8–10], a process possibly similar to the biogenesis of lysosome-related organelles [9]. Acid phosphatase activity was detected in immature secretory granules, suggesting that this enzyme is required for processing of secretory proteins [11]. Secretory granules can fuse with lysosomes, which regulates the glue granule pool by selective degradation (crinophagy) [12, 13]. The molecular participants of the secretory granule-lysosome fusion in the *Drosophila* salivary gland include factors mediating the fusion of lysosomes with other organelles, too [12, 14]. The lysosomal system may also play other roles in the dynamics of secretory granules. For instance, acidification and chloride ion uptake of the insulin granules were shown to be required for exocytosis in pancreas beta cells [15]. Recently, the lowering of granular pH accompanied by specific changes in calcium and chloride ion concentration has also been shown to be required for normal secretory granule maturation in *Drosophila* [16]. Moreover, uncontrolled secretion of granules (enlarged partly due to fusions with lysosomes) can be observed in the secretory glands of TRPML1 (a lysosomal Ca^2+^-permeable cation channel) mutant mice developing mucolipidosis type IV [17]. This suggests a role for secretory granule size and lysosomal fusion in acquiring competence for secretion.

The small GTPase Rab26 was first described from a rat pancreatic cDNA library [18]. It belongs to the type III Rab GTPases, which play a role in exocytosis-related intracellular vesicular transport along with Rab3 and Rab27 isoforms and Rab37 [19]. Rab26 was found on the surface of secretory granules in the acinar cells of parotid glands in rats [20, 21], along with Rab3D and Rab27, and Rab26 was shown to regulate amylase secretion [20]. MIST1 transcription factor and its targets including Rab3D and Rab26 were described as crucial factors for secretory granule maturation in gastric zymogenic cells [22]. In contrast, Rab26 on the surface of insulin granules was found to restrict insulin secretion in the pancreas [23]. Human Rab26 was also found on Lamp1- and Cathepsin D-positive lysosomes, and Rab26 expression resulted in a perinuclear redistribution of lysosomes [24]. Based on these, Rab26 appears to be an important regulator of secretory granule dynamics in different secretory cell types, although its exact roles are unclear.

Rab26 is also reported to play important roles in the nervous system. Rab26 overexpression resulted in the clustering of synaptic vesicles in mammalian neurons, which were positive for Atg16L1, LC3B and Rab33B; moreover, GTP-bound Rab26 directly binds to Atg16L1 [25], an interaction which has been confirmed by other studies [26, 27]. This suggests a connection between Rab26 and autophagic degradation of synaptic vesicles. In a recent study, null mutant lines were generated for all 26 *Drosophila* Rab genes [28]. The Rab26 null mutant flies are viable and develop normally; however, stimulus-dependent neuronal function defects were detected, and this role of Rab26 appears to be independent of autophagy [28]. A role for Rab26 has been implicated in different pathological conditions such as cancer [27, 29], acute lung injury [26], pulmonary microvascular endothelial hyperpermeability and apoptosis [30] and *Coxiella* pathogenesis [31]; thus, clarifying its functions would be important for better understanding of the molecular background of these processes.

The contradictory role of Rab26 in secretory granule maturation led us to investigate it in the salivary gland of *Drosophila*, an established model for the study of secretion. Here we demonstrate that *Drosophila* Rab26 regulates the early steps of secretory granule maturation, and it delays acidification and exocytosis until they reach proper size via homotypic fusions.

## Results

### Rab26 localizes to small secretory granules

**Fig. 1:**
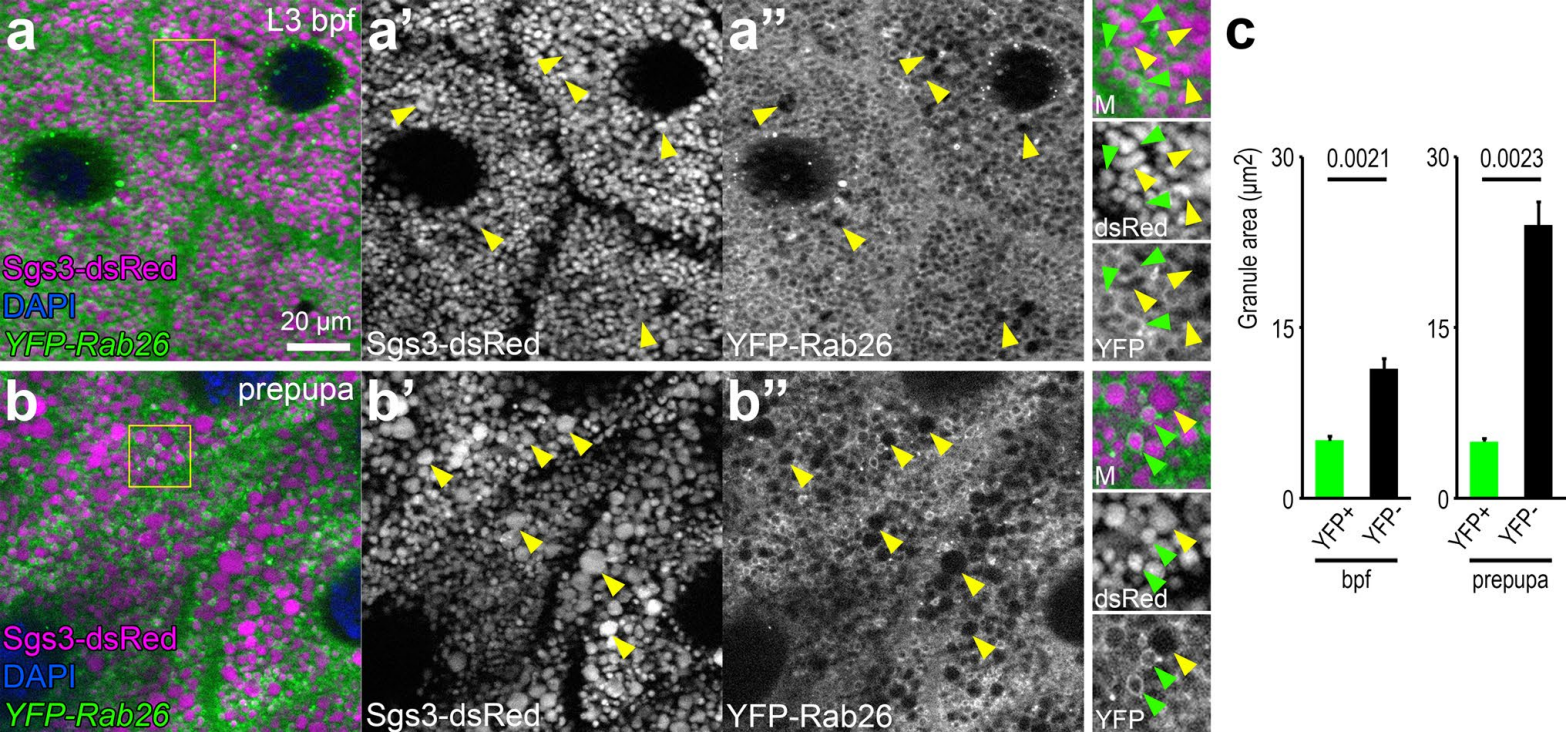
Rab26 localizes to small secretory granules. **a**, **b** YFP-Rab26 forms clear rings around the smaller secretory granules (green arrowheads) in the salivary gland cells at the late L3 stage before and during puparium formation (**a**, **b**), but not around the larger granules, which are detected in increased numbers at the prepupal stage (yellow arrowheads) (**a**, **b**). Sgs3-dsRed marks secretory granules. Insets show merged images (top, M), Sgs3-dsRed channels (middle) and YFP-Rab26 channels (bottom) with a 2 × magnification enlarged from the boxed areas of the representative main panels (**a**, **b**). DAPI marks nuclei. **c** Quantification of data shown in **a**, **b**; *n* = 50 randomly selected granules from 10 cells from 4 animals. Error bars denote SE and the numbers above the clasps show *p* values. *Bpf* before puparium formation. Scale bar, 20 µm (**a**, **b**)

Rab26 has been found to localize on the surface of secretory granules in the acinar cells of rat parotid glands [20, 21]. We thus tested whether *Drosophila* Rab26 is also found on glue granules in salivary gland cells. A dsRed-tagged, truncated form of Sgs3 (salivary gland secretion 3) protein was expressed from its own regulatory sequences [2, 12] to label glue granules, and a Gal4-responsive *UAS* promoter-driven, YFP-tagged form of Rab26 [32] was expressed by *fkh*-Gal4 in the salivary gland. Just before puparium formation, many of the glue granules are already mature and ready for secretion. At this stage, YFP-Rab26 rings were detected around most of the smaller glue granules but were absent from larger vesicles (Fig. 1a), which was even more obvious in prepupal cells (Fig. 1b). Quantification of granule sizes clearly showed that YFP-Rab26 associates with smaller granules at both developmental stages (Fig. 1c). This is also the case in wandering L3 larvae expressing YFP-Rab26 (Fig. S1a, d).

We also expressed *YFP-Rab26[Q250L]* encoding a point mutant protein defective in GTP hydrolysis [32]. GTP-locked Rab26 persisted on enlarged secretory granules (Fig. S1b, e). These results indicate a role for Rab26 in secretory vesicle maturation, and its dissociation appears to be important for enabling the later steps of glue granule maturation.

We confirmed punctate endogenous Rab26 expression in salivary gland cells using a previously published EYFP-Rab26 knockin line [33] (Fig. S1f).

### Rab26 counteracts secretory granule acidification

**Fig. 2:**
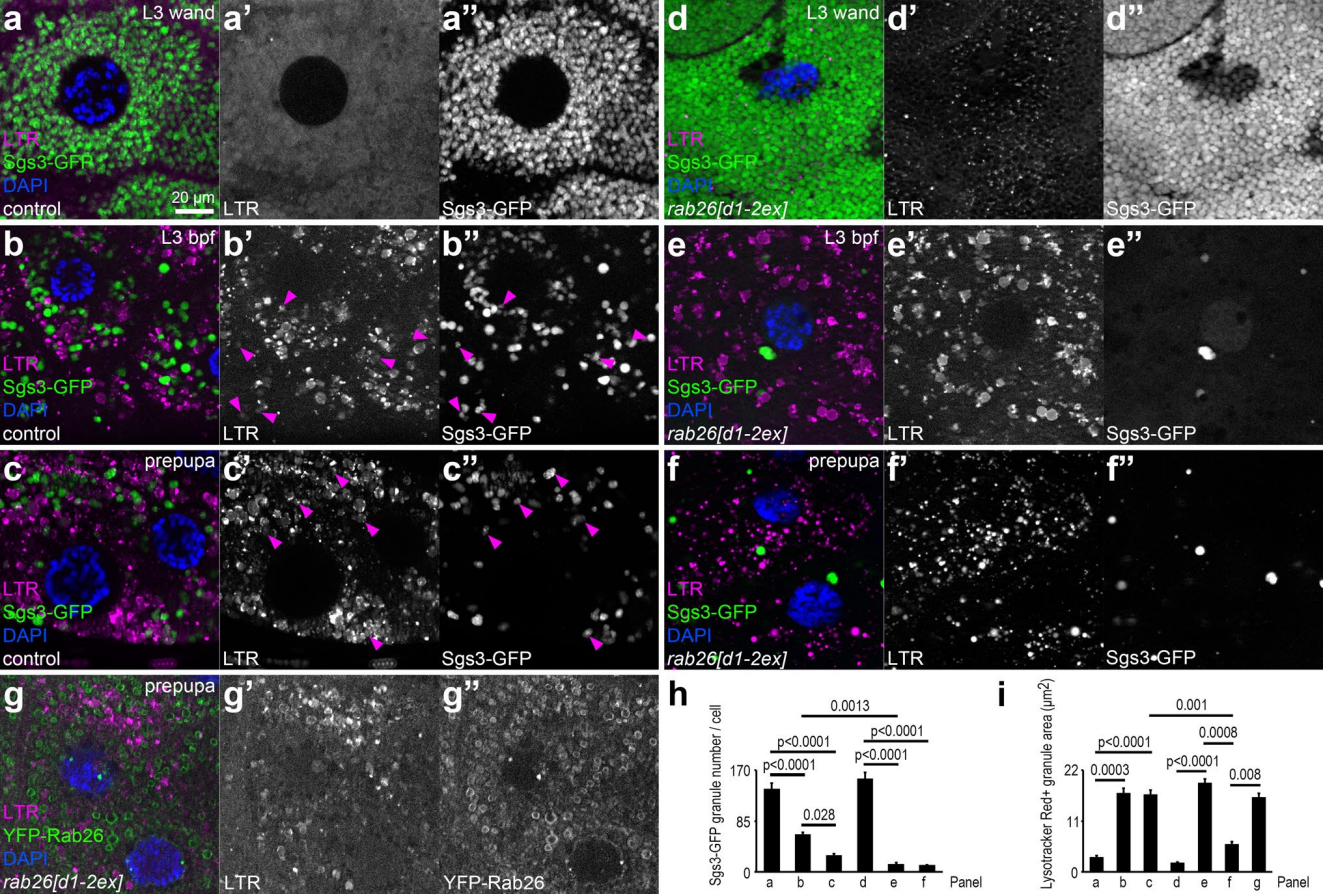
*rab26* knockout accelerates secretory granule acidification. **a–c** Lysotracker Red (LTR) signal is scarce in the salivary gland cells of wandering control larvae, when mostly Sgs3-GFP-positive secretory granules are present (**a**). Sgs3-GFP signal gradually decreases due to its sensitivity to the acidic environment, concomitant with the appearance of large LTR-positive structures before puparium formation and in prepupae (**b**, **c**). **d–f** LTR does not stain GFP + granules in *rab26* knockout salivary gland cells of wandering larvae, but it already labels many small dots throughout the cell (**d**). Large LTR-positive granules appear and Sgs3-GFP + glue granules are practically absent in *rab26* mutant cells shortly before puparium formation (**e**). Punctate LTR signal is seen in the prepupal salivary gland of *rab26* mutants (**f**). **g** YFP-Rab26 expression rescues the LTR phenotype of *rab26* knockout. Magenta arrowheads show granules with faint Sgs3-GFP signal and weak LTR staining (**b**, **c**). DAPI marks nuclei. **h, i** Quantification of data shown in **a**–**g**; *n* = 10 cells from 4 animals (**h**), *n* = 100 randomly selected granules from 10 cells from 4 animals (**i**). Error bars denote SE and the numbers above the clasps show *p* values. *Wand* wandering, *bpf* before puparium formation. Scale bar, 20 µm (**a**–**g**)

Acidification of insulin granules during their maturation before exocytosis appears to be important in pancreatic beta cells [15]. Acidification also appears to promote restructuring of glue granule contents in *Drosophila* [16]. We thus decided to find out whether Rab26 plays a role in secretory granule acidification. We tested this by staining salivary gland cells expressing a green fluorescent protein (GFP)-tagged, truncated Sgs3 protein [6] with Lysotracker Red, a vital dye that marks acidic structures. We detected practically no Lysotracker signal in the granules that all exhibited strong GFP signal in control wandering L3 stage larvae (Fig. 2a). Before puparium formation, the fluorescence of Sgs3-GFP starts to decrease because of quenching due to a drop of pH within glue granules [12], which was accompanied by the appearance of large Lysotracker Red-positive organelles that exhibited no or weak GFP fluorescence, likely marking the mature, acidic granules (Fig. 2b). This tendency continued in the prepupal salivary gland: While some GFP-positive structures remained, many large acidic organelles with no or faint GFP signal were observed (Fig. 2c).

We next used a recently published Rab26 null mutant line, where the first two exons of the *Rab26* gene are replaced by a *3xP3-dsRed* cassette [28] (hereafter referred to as *rab26[d1-2ex]*) for analyzing the phenotype of *rab26* knockout cells. While mutant salivary gland cells also contained many small Sgs3-GFP granules at the wandering L3 stage, small Lysotracker Red dots were already obvious in *rab26* mutants unlike in controls (Fig. 2d). Just before puparium formation, *rab26[d1-2ex]* cells also contained large Lysotracker-positive organelles similar to controls, while much fewer GFP-positive granules could be observed in mutants (Fig. 2e). GFP-positive granules were again scarce in mutant prepupae, and Lysotracker Red marked numerous smaller punctae in mutant cells rather than large granules as seen in controls (Fig. 2f). These smaller acidic structures are possibly the remnants of non-exocytosed glue granules degraded by crinophagy [12], or related acidic lysosomes. YFP-Rab26 expression rescued the aberrant Lysotracker phenotype of *rab26[d1-2ex]* mutants at the prepupal stage, as the size of acidic structures matched that in control prepupal salivary glands (Fig. 2g). Quantification of data verified that the loss of GFP signal is accelerated and the larger Lysotracker Red-positive granules disappear by the prepupal stage in *rab26[d1-2ex]* animals, which is rescued by Rab26 reexpression (Fig. 2h, i), and statistically significant increases of Lysotracker dot numbers are detected during normal development and in *rab26* mutant versus control wandering animals (Fig. S2f). These indicate that loss of Rab26 accelerates glue granule acidification.

We also quantified glue granule sizes of control, *rab26* knockout and Rab26-overexpressing salivary gland cells, which showed that granules are often larger in the mutants than in the controls (Fig. S2a). However, most granules remain small in Rab26-overexpressing cells, suggesting that Rab26 restricts the granules from the later steps of maturation (Fig. S2a).

To further confirm that the Lysotracker Red-positive, acidified structures are mature glue granules, we stained salivary glands with Lysotracker Green (LTG) and analyzed its colocalization with the more pH-resistant Sgs3-dsRed. While there is almost no LTG signal in the salivary gland cells at the wandering stage (Fig. S2b), extensive colocalization is detected at later stages (Fig. S2c–e), indicating that glue granules indeed acidify during their maturation.

**Fig. 3:**
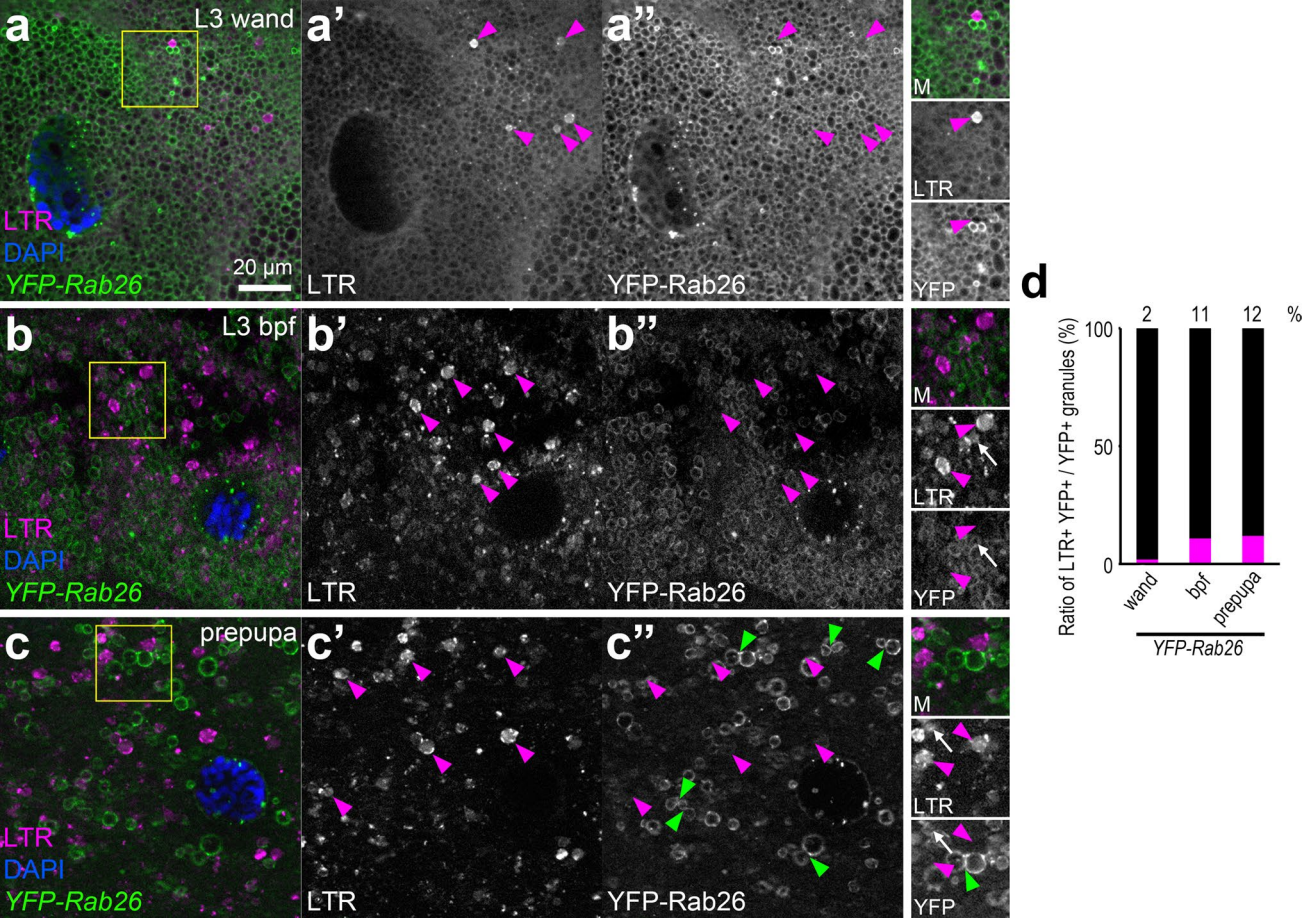
Rab26 inhibits the acidification of secretory granules. **a–c** YFP-Rab26 shows ring-like localization marking small secretory granules in the salivary gland of wandering larvae. Some LTR-positive structures also appear, but they are not surrounded with YFP-Rab26 (**a**). YFP-Rab26-positive secretory granules still never overlap with the more abundant LTR signal at later stages (**b**, **c**). Several of the YFP-Rab26 vesicles seem to be in close contact with each other (green arrowheads) (**c**). Magenta arrowheads show LTR-positive structures that are mostly not surrounded with YFP-Rab26 (**a**–**c**). White arrows show rarely occurring colocalizing structures (**b**, **c**). Insets show merged images (top, M), Lysotracker Red channels (middle, LTR) and YFP-Rab26 channels (bottom) with a 1.3 × magnification enlarged from the boxed areas of the representative main panels (**a**–**c**). DAPI marks nuclei. **d** Quantification of data shown in **a**–**c**; *n* = 100 randomly selected granules from 10 cells from 4 animals. *Wand* wandering, *bpf* before puparium formation. Scale bar, 20 µm (**a**–**c**)

We also performed Lysotracker Red staining on larval salivary glands expressing YFP-Rab26. There were only a few acidic structures, which did not overlap with the YFP-Rab26 rings in wandering L3 larvae (Fig. 3a). At later stages, while larger acidic organelles formed, most of the YFP-Rab26-positive granules still did not exhibit acidification (Fig. 3b, c). Quantification of data also showed that only a small subset of Rab26-positive granules could acidify (Fig. 3d). Closely apposed YFP-Rab26 vesicles were often seen, possibly marking homotypic fusion events between maturing glue granules (Fig. 3c). These data further support that Rab26 is a negative regulator of secretory granule acidification.

### *rab26* knockout accelerates secretory granule maturation

**Fig. 4:**
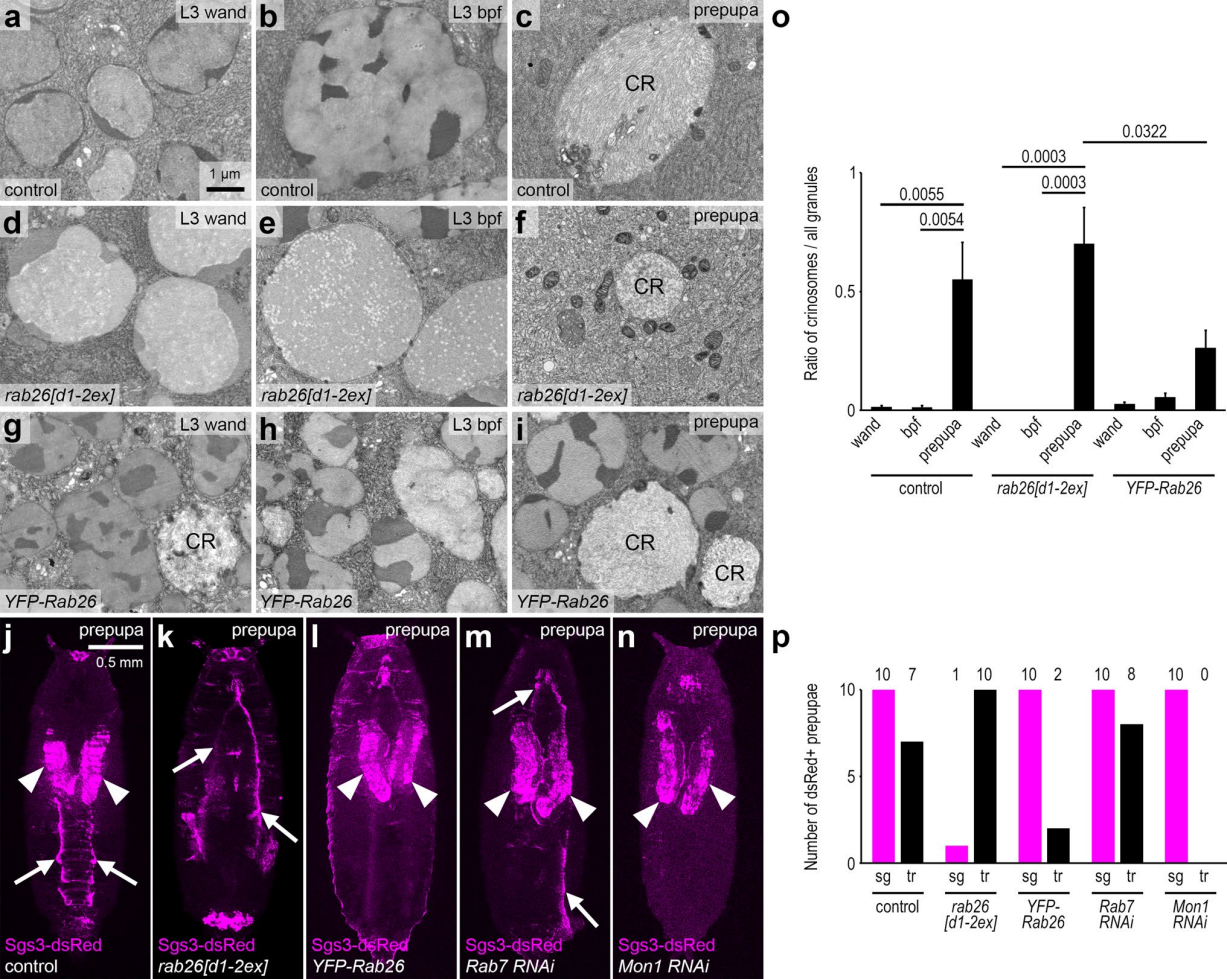
Rab26 inhibits secretory granule maturation and release. **a–c** Electron micrographs of salivary glands from L3 stage wandering larvae show large numbers of relatively small secretory granules (**a**). Fewer and larger granules are seen shortly before puparium formation (**b**). Large crinosomes appear in the salivary gland cells of prepupae (**c**). **d–f** *rab26* null mutation results in premature secretory granule maturation. Compared to the control, the granules are larger already at the wandering stage (**d**), while at the end of the L3 stage, mainly mature granules are seen, ready for release (**e**). Only small crinosomes are present in the prepupal salivary gland cells of mutant animals (**f**). **g–i** Overexpression of wild-type Rab26 appears to inhibit secretory granule maturation as the large numbers of small, immature granules present in the salivary gland of wandering larvae (**g**) generally fail to reach the size and maturity of those observed in controls by the end of the L3 stage (**h**). Many immature granules are still present even in prepupae, while crinosomes also appear by this time (**i**). **j–n** Glue can still be detected in the salivary gland lobes marked by the Sgs3-dsRed reporter in control prepupae (white arrowheads), and traces of secreted glue are also visible on the ventral surface of prepupae (white arrows) (**j**). Deposited glue is clearly visible, while Sgs3-dsRed is absent from the salivary glands of *rab26* mutant prepupae, indicating more complete glue release and/or degradation of residual glue in *rab26* mutants (**k**). Rab26 overexpression inhibits the release of glue from the salivary glands of prepupae, as Sgs3-dsRed is only seen in the glands and no traces on the outer surface can be detected (**l**). Rab7 knockdown has no effect on glue distribution (**m**), while silencing of Mon1 prevents proper glue release: only the glands are labeled by Sgs3-dsRed, and no deposits are seen (**n**). **o** Quantification of data shown in **a**–**i**; all detected granules were quantified in each case (*n* = 267 (**a**), 333 (**b**), 16 (**c**), 494 (**d**), 214 (**e**), 14 (**f**), 473 (**g**), 415 (**h**), 349 (**i**) granules from 5 salivary glands in each case). Error bars denote SE and the numbers above the clasps show *p* values. **p** Quantification of data shown in (**j**–**n**); 10 prepupae were evaluated per genotype. Numbers above the graph show how many prepupae had signal in the salivary gland (sg) and traces on the outer prepupal surface (tr). *CR* crinosome, *wand* wandering, *bpf* before puparium formation. Scale bar, 1 µm (**a**–**i**) and 1 mm (**j**–**n**)

The mucoprotein content of the granules is gradually reorganized during glue granule maturation, which is clearly visible under the electron microscope. Since this process appears to depend on acidification [16], we performed ultrastructural analysis of *rab26* mutants. Small secretory granules with a typical diameter of 2–3 µm appear in large numbers in control salivary gland cells at the wandering L3 stage (Fig. 4a). Glue granules greatly enlarge just before their exocytosis at the time of puparium formation (Fig. 4b). Secretion takes place in a relatively short time, after which the cells contain much fewer residual secretory granules. These then undergo lysosomal degradation via crinophagy (Fig. 4c, o) [4, 12]. The ultrastructure of these so-called crinosomes significantly differs from that of the intact granules, as the lumenal content is disintegrated during the degradation process.

We observed that granules are enlarged in *rab26[d1-2ex]* mutant cells at the wandering L3 stage, and based on their ultrastructure, these were ahead of controls in the maturation process (Fig. 4d). Just before pupariation, large granules—ready for release—could mainly be observed (Fig. 4e). The ultrastructure of their content was very similar to the appearance of the glue secreted into the lumen of the gland, suggesting that these are bona fide secretory granules and not crinosomes (Fig. S3a, b). Secretion appeared to be mostly completed by this stage in mutants (Fig. S3a, b). We found hardly any secretory granules in the prepupal salivary gland cells of *rab26* mutants, only small crinosomes along with occasionally appearing multivesicular bodies and electron-dense lysosome-like organelles (Fig. 4f, o). These likely represent the small Lysotracker-positive structures seen in fluorescent microscopy (Fig. 2f).

We also investigated the ultrastructure of salivary glands overexpressing *YFP-Rab26*. Many small, immature glue granules were detected throughout the cytosol in wandering L3 stage larvae (Fig. 4g). Interestingly, some relatively small structures resembling crinosomes were also present, suggesting that the restricted maturation of the granules could lead to early crinophagic activation, possibly in order to remove a subset of the accumulated granules. Surprisingly, secretory granules remained smaller and denser even shortly before pupariation, and the restructuring of their content appeared to be attenuated (Fig. 4h). Nevertheless, some enlarged granules with a mature appearance could also be detected at this stage, which likely represent the acidic organelles seen by Lysotracker Red staining of the YFP-Rab26-expressing salivary glands (Fig. 3b). Strikingly, the vast majority of the granules was still immature at the prepupal stage, while crinosomes were also present (Fig. 4i, o). These results suggest that Rab26 counteracts the later steps of glue granule maturation, likely by negatively regulating their acidification.

We next investigated how Rab26 influences the secretion of glue granules by analyzing the distribution of Sgs3-dsRed at the organismal level in prepupae. Traces of secreted glue on the ventral surface of immobile animals were obvious in controls, while strong signal could be still detected in the salivary glands (Fig. 4j, p). Strikingly, fluorescent signal was practically missing from the salivary glands and only the deposited glue was seen in the case of *rab26[d1-2ex]* mutants, based on dsRed signal on the ventral surface of prepupae (Fig. 4k, p). On the contrary, salivary gland-specific overexpression of YFP-Rab26 prevented glue from leaving the salivary glands (Fig. 4l, p). Since secretion itself was not blocked from the cells into the lumen even in case of GTP-locked YFP-Rab26[Q250L] expression (Fig. S3c), Rab26 seems to inhibit the release of glue from the lumen. Thus, we hypothesize that the structure or viscosity of the glue is altered by Rab26 overexpression.

**Fig. 5:**
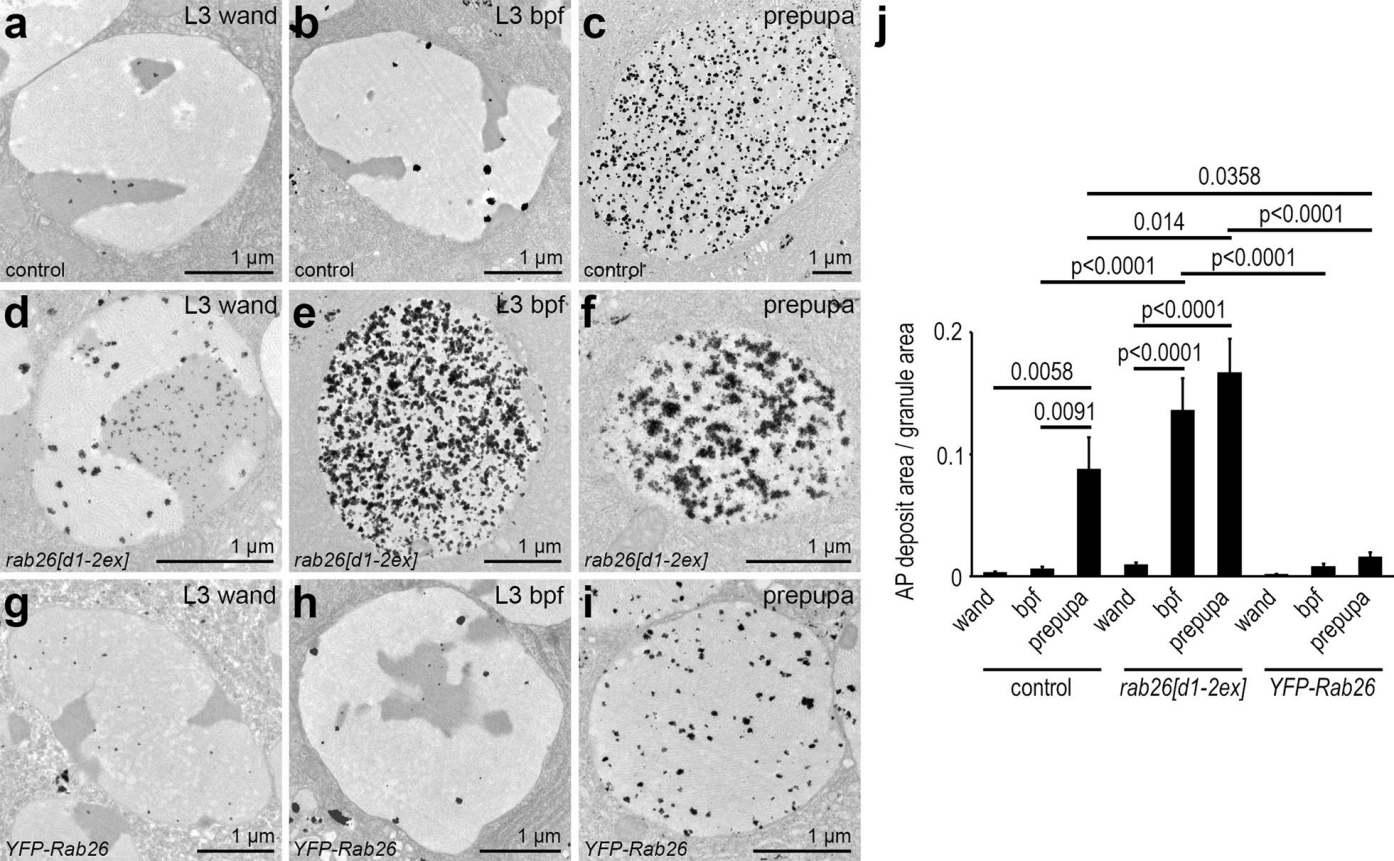
*rab26* knockout accelerates acid phosphatase delivery to secretory granules. **a–c** Gömöri’s acid phosphatase (AP) assay detects only traces of enzyme activity in the glue granules of control wandering L3 larvae (**a**). This is mildly increased shortly before pupariation (**b**) and becomes strongly elevated in the mature granules of prepupal gland cells (**c**). **d–f** Secretory granule maturation is accelerated in *rab26* mutants as more reaction product can be detected in the granules at all examined stages (**d**–**f**). Please note that the few remaining secretory granules contain even more reaction product at later stages, indicating elevated AP activity (**e**, **f**). **g–i** Overexpression of YFP-Rab26 does not perturb acid phosphatase delivery to secretory granules as the enzyme reaction product is present in the granules both at wandering L3 stage and shortly before pupariation (**g** and **h**, respectively), to a similar extent seen in the control cells. However, glue granules exhibit less acid phosphatase activity than those seen in control prepupae, indicating a delay in granule maturation (**i**). **j** Quantification of data shown in **a**–**i**; n = 10 randomly selected granules from 5 salivary glands in each case. Error bars denote SE and the numbers above the clasps show *p* values. *Wand* wandering, *bpf* before puparium formation. Scale bars, 1 µm (**a**–**i**)

We next performed acid phosphatase enzyme cytochemistry on salivary glands as an additional proxy for secretory granule acidification. Immature glue granules showed very little activity in ultrastructural images of control cells (Fig. 5a, j), which seemed to only slightly increase during their maturation (Fig. 5b, j). Of note, residual large granules exhibited strong acid phosphatase activity in the prepupal salivary gland cells (Fig. 5c, j), as described before [12]. In case of *rab26[d1-2ex]* mutants, salivary gland cells contained glue granules with seemingly increased amounts of reaction products in wandering L3 stage larvae (Fig. 5d, j). Interestingly, the distribution of the signal was distinct in the different structural domains of the granules: the more electron-dense parts exhibited smaller deposits of reaction products, and these were more dispersed than the bigger but fewer deposits seen in less electron dense parts. At later stages, the few remaining granules showed very robust acid phosphatase reactivity (Fig. 5e, f, j). Rab26 overexpression did not block acid phosphatase activity in the granules, as the pattern of the reaction products appeared similar to the control before pupariation (Fig. 5g, h, j). We again saw mainly maturing secretory granules instead of crinosomes at the prepupal stage, and YFP-Rab26 expression led to a statistically significant reduction in acid phosphatase reactivity when compared to control and *rab26* mutant prepupae (Fig. 5i, j). Taken together, *rab26* knockout strongly increases acid phosphatase activity in glue granules likely because of accelerated maturation.

### Rab26 loss enhances lysosomal transport to secretory granules

**Fig. 6:**
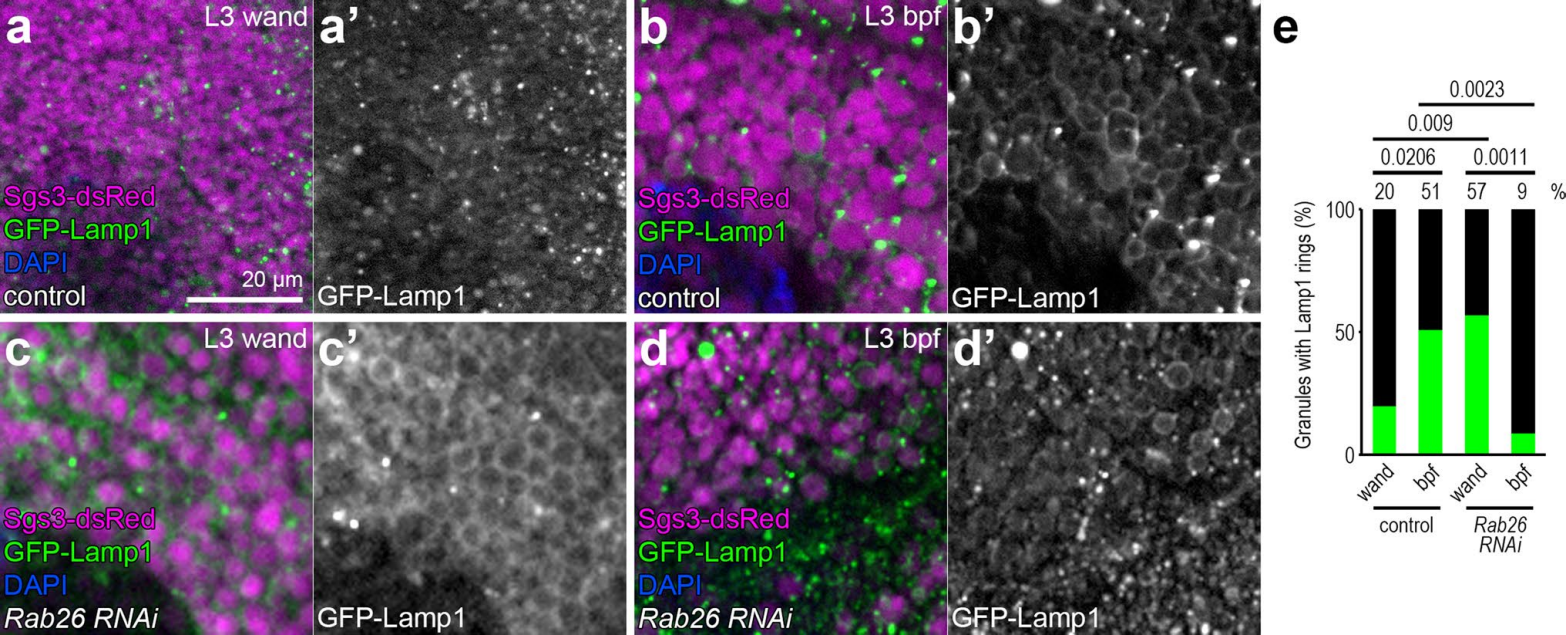
Knockdown of Rab26 accelerates lysosomal transport to secretory granules. **a–d** Only a few Sgs3-dsRed-positive secretory granules are surrounded by GFP-Lamp1 rings in the salivary gland cells of late L3 stage control larvae (**a**), while many of the granules become positive for GFP-Lamp1 shortly before puparium formation (**b**). Lysosomal transport appears to take place earlier in *rab26* silenced salivary gland cells because large GFP-Lamp1 rings are already abundant in the wandering larvae (**c**), and they are found in smaller numbers than in control cells by the time this peaks in controls (**d**). DAPI marks nuclei. **e** Quantification of data shown in **a**–**d**; *n* = 100 randomly selected granules from 10 cells from 4 animals. The numbers above the clasps show *p* values. *Wand* wandering, *bpf* before puparium formation. Scale bar, 20 µm (**a**–**d**)

Secretory granules acquire the lysosomal membrane reporter Lamp1 shortly before pupariation [12, 34]. We used this reporter to assess how Rab26 influences lysosomal transport to the secretory granules. Only a few Lamp1-positive granules were seen in the salivary gland cells of control wandering L3 larvae (Fig. 6a, e), while this reporter readily formed rings around most Sgs3-dsRed-positive vesicles later on (Fig. 6b, e), as described [12]. Interestingly, more than half of the granules were surrounded by Lamp1 rings already at the wandering stage upon silencing of Rab26 (Fig. 6c, e) and only a few rings remained shortly before pupariation (Fig. 6d, e). To confirm the efficacy of Rab26 RNAi knockdown, we co-expressed YFP-Rab26[Q250L] with Rab26 RNAi, which indeed abolished the YFP signal (Fig. S1c). Taken together, we conclude that Rab26 functions to inhibit the transport of lysosomal contents to secretory granules, likely to retain glue granules from acidification and maturation.

### Rab26 is not a general inhibitor of lysosomal fusions

The most important small GTPases needed for lysosomal fusions are Rab2, Rab7 and Arl8. These were all found to be important for three different types of cargo delivery to lysosomes: endosome-, autophagosome- and secretory granule-lysosome fusions [12, 14, 35–40]. These observations motivated us to examine whether *Drosophila* Rab26 influences cargo flux through these other lysosome-related pathways during autophagosome or endosome maturation.

We thus performed western blot analysis of the level of lipidated, autophagosome-associated Atg8a-II that is transported to the lysosome by fusion [41, 42]. We saw no obvious difference between controls and *rab26* null mutants or animals expressing Rab26 RNAi or GTP-locked or GDP-locked forms in all cells, respectively (Fig. S4a, b). To further test this, we turned to confocal microscopy of mosaic fat tissues from starved early L3 larvae that express a genomic promoter-driven 3xmCherry-Atg8a reporter in all cells, and GFP-Lamp1 in cell clones. This system allowed us to examine autophagosome–lysosome fusion since 3xmCherry-Atg8a is transported to Lamp1-positive lysosomes if fusion is successful [14]. We detected colocalizing structures representing autolysosomes in both control and Rab26 knockdown cells, and the size and distribution of these vesicles were indistinguishable (Fig. S4c-e). These results suggest that Rab26 does not play an important role in autophagosome maturation.

Garland nephrocytes serve as an excellent model to study the endocytic pathway in *Drosophila*. These cells continuously filter the hemolymph and transport the endocytosed material to lysosomes through Rab5-positive early and Rab7-positive late endosomes [43, 44]. We immunostained garland nephrocytes of wandering third-instar larvae for endogenous Rab5 and Rab7 to examine whether Rab26 manipulation perturbs endosomal compartments. Rab5 marks early endosomes that are present as a layer of small vesicles under the plasma membrane, while Rab7 is found deeper inside the cells on the larger late endosomes (Fig. S5a). Neither *rab26* null mutation nor Rab26 knockdown altered the appearance of Rab5 or Rab7 signals (Fig. S5b, c, f). The overexpression of wild-type or GTP-locked YFP-Rab26 did not perturb Rab7-positive late endosome size or distribution either and the YFP signal was diffuse throughout the cytosol in both cases (Fig. S5d–f), indicating that Rab26 only associates with secretory vesicles and not with endosomes. Thus, we concluded that Rab26 is dispensable for endolysosomal trafficking in *Drosophila* nephrocytes.

A subset of fruit fly genes, whose mutations cause visible alterations of eye color, functions in vesicular transport to pigment granules, which are lysosome-related organelles in the pigment cells of compound eyes [45]. We tested whether Rab26 knockdown or overexpression alters eye pigmentation. Neither Rab26 silencing nor the overexpression of wild-type or GTP-locked Rab26 perturbed eye color (Fig. S6a–d), suggesting that Rab26 does not play a critical role in pigment granule biogenesis.

Taken all these data together, we conclude that *Drosophila* Rab26 is not a general regulator of lysosomal fusions.

### Mon1-dependent removal of Rab26 during secretory granule maturation

**Fig. 7:**
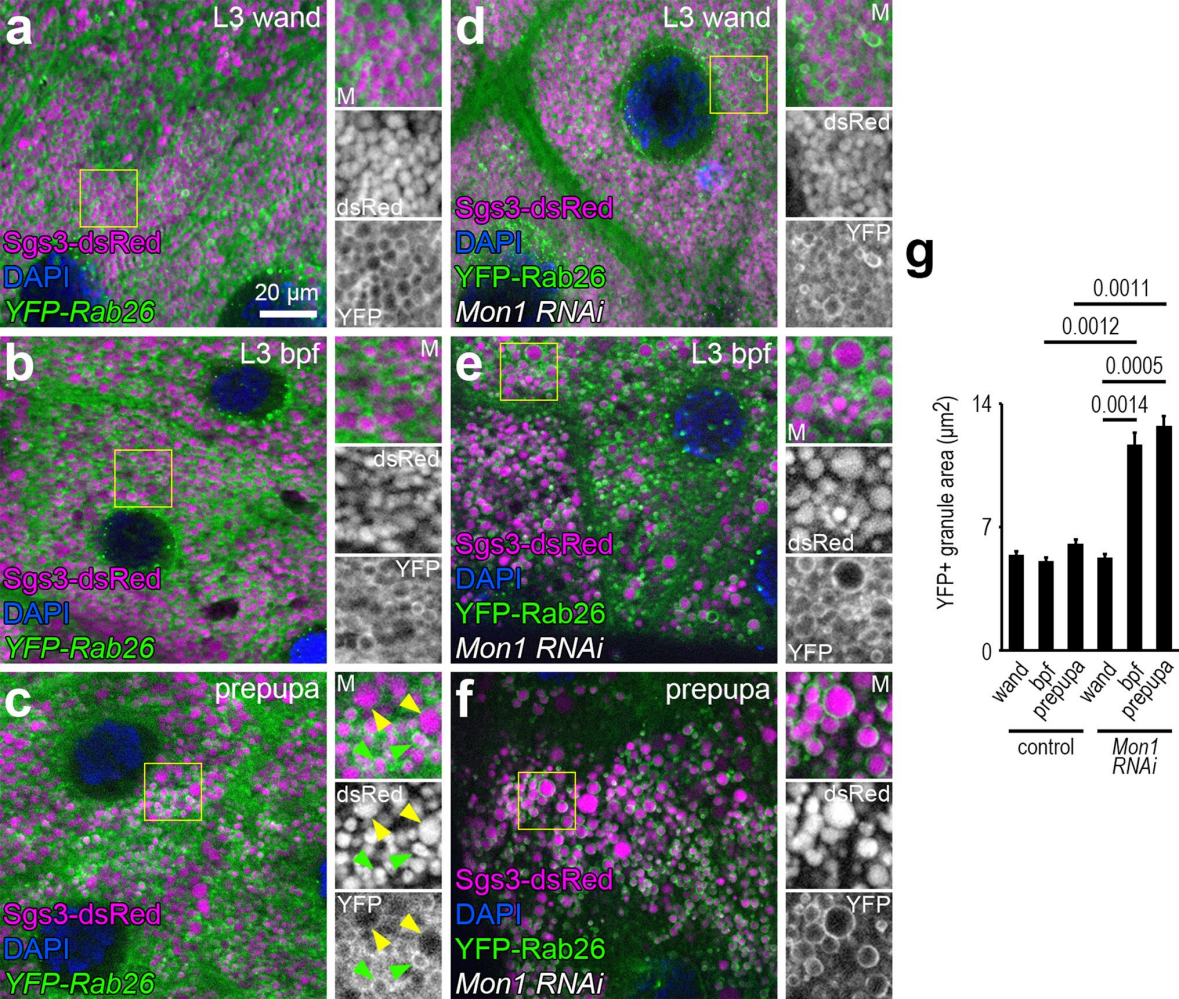
Mon1 knockdown inhibits the dissociation of Rab26 from secretory granules. **a–c** YFP-Rab26 is present on the smaller secretory granules in the salivary gland cells of all examined larval stages (**a**–**c**), but not on the larger ones, which are mainly observed in the prepupae (**c**) (marked by green and yellow arrowheads, respectively, in panel **c**). **d–f** Mon1 knockdown inhibits the dissociation of YFP-Rab26 from glue granules as Rab26 is now also seen surrounding the larger granules at different larval stages (**d**–**f**). Mon1 silencing also increases the average granule size from the end of the late L3 stage (**e**, **f**). Sgs3-dsRed marks the secretory granules. Insets show merged images (top, M), Sgs3-dsRed channels (middle) and YFP-Rab26 channels (bottom) with a 2 × magnification enlarged from the boxed areas of the representative main panels (**a**–**f**). DAPI marks nuclei. **g** Quantification of data shown in **a**–**f**; *n* = 100 randomly selected granules from 10 cells from 4 animals. Error bars denote SE and the numbers above the clasps show *p* values. *Wand* wandering, *bpf* before puparium formation. Scale bar, 20 µm (**a**–**f**)

Rab7 is required for crinophagy in the *Drosophila* salivary gland [12]. We hypothesized that Rab26 could be replaced by Rab7 on those glue granules that will eventually be degraded. We thus investigated whether the Rab7 activator Mon1 [35, 40] may play a role in displacing Rab26 from maturing glue granules by silencing Mon1 in salivary gland cells. As shown also in Fig. 1, YFP-Rab26 forms rings around the smaller secretory granules, and larger granules lacking YFP-Rab26 appear towards the prepupal stage (Fig. 7a–c). Strikingly, Rab26 was retained on the surface of large glue granules upon Mon1 knockdown (Fig. 7d–g). Even YFP-Rab26 rings were more pronounced, and the background signal seemed to decrease in Mon1-silenced cells. Based on these data, we conclude that the Rab7 activator Mon1 is critical for removing Rab26 from mature secretory granules.

**Fig. 8:**
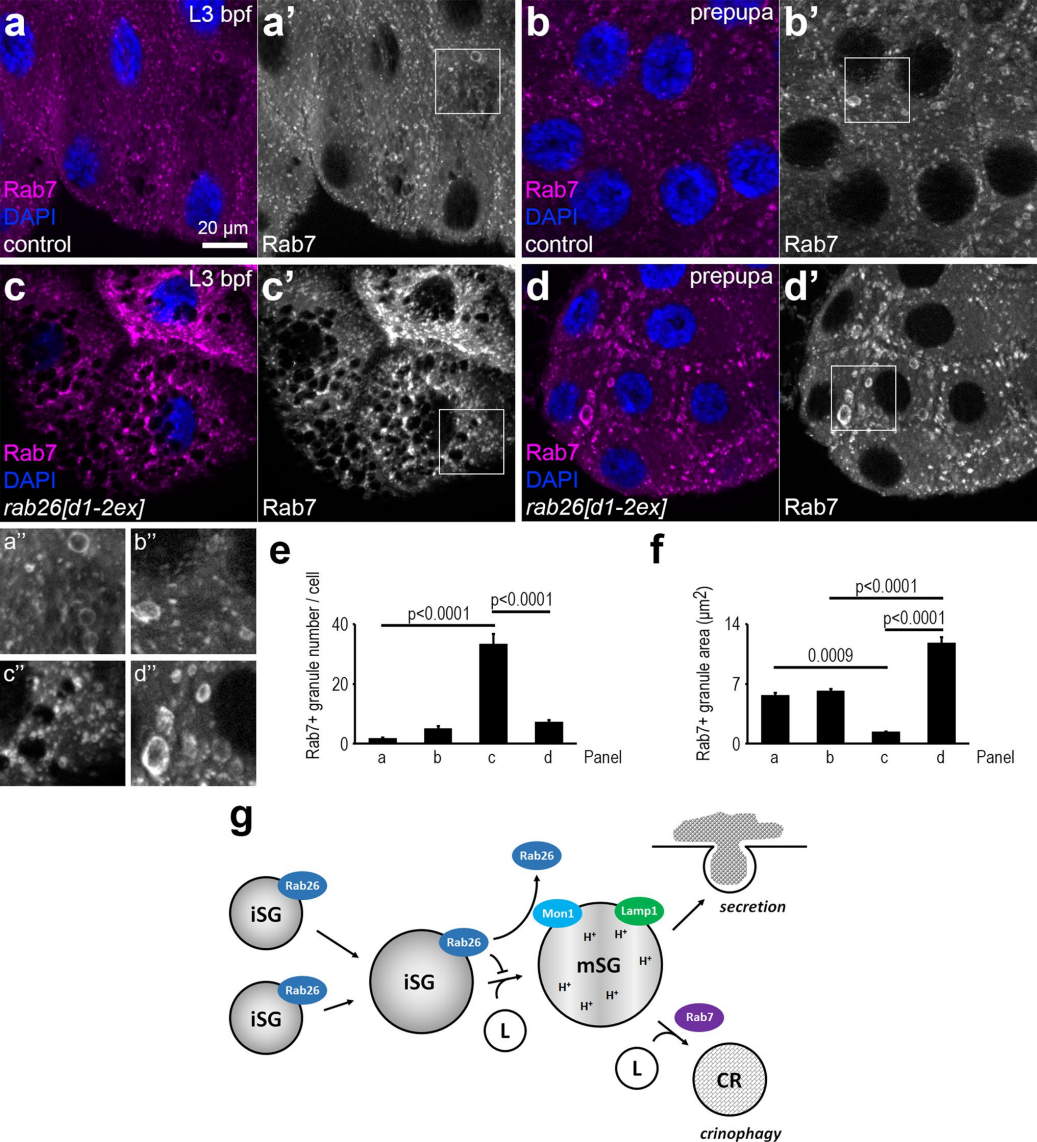
Knockout of *rab26* enhances Rab7 vesicular localization. **a**, **b** Immunostainings reveal only a few Rab7 rings before pupariation in control salivary gland cells (**a**) and similar numbers and sizes of Rab7-positive structures are detected in control prepupae (**b**). **c**, **d** Large numbers of very small Rab7-positive vesicles are frequently observed before puparium formation in *rab26* mutants (**c**), while larger Rab7 structures develop in smaller numbers in the prepupal salivary glands (**d**). Insets show Rab7 channels (a”–d”) with a 2 × magnification enlarged from the boxed areas of the main panels (a’–d’). DAPI marks nuclei. **e–f** Quantification of data shown in **a**–**d**; *n* = 10 cells from 4 animals (**e**), *n* = 100 randomly selected granules from 10 cells from 4 animals (**f**). Error bars denote SE and the numbers above the clasps show *p* values. **g** A model depicting the role of Rab26 in glue secretion. During glue granule biogenesis, Golgi-derived, immature glue containing vesicles fuse with each other to form larger granules. Rab26 temporarily restricts glue granules from further steps of maturation, possibly to provide time for reaching the proper size and/or composition. Later, Rab26 dissociates in a Mon1-dependent manner, and glue granules fuse with lysosomes to become acidic. Most of the mature glue granules are eventually secreted, but some of them remain inside the cells. These are then removed by Rab7-mediated lysosomal degradation (crinophagy). *Bpf* before puparium formation, *iSG* immature secretory granule, *mSG* mature secretory granule, *L* lysosome, *CR* crinosome (glue granule degrading lysosome). Scale bar, 20 µm (**a**–**d**)

We next analyzed the localization of endogenous Rab7. Shortly before or after secretion, Rab7 rings could be detected besides its punctate pattern throughout the cytosol (Fig. 8a, b), likely marking endosomes [7, 46] and glue granules to be degraded by crinophagy [12]. Interestingly, high numbers of very small Rab7-positive structures were observed alongside large Rab7-negative structures likely representing mature glue granules before secretion in *rab26* mutant cells (Fig. 8c), whereas there were bigger but fewer Rab7 vesicles in mutant prepupae (Fig. 8d), suggesting that they had fused with each other by this time. Quantification of data shows that Rab26 loss significantly influences the number and size of Rab7-positive organelles in the salivary gland cells (Fig. 8e, f).

Finally, we sought to confirm the functional relevance of Mon1 and/or Rab7 in removing Rab26 from secretory granules using the glue release assay. We expected that loss of Mon1 should also prevent proper glue deposition during puparium formation because Rab26 is retained on the surface of large glue granules. Indeed, salivary gland-specific knockdown of Mon1 using a previously validated RNAi line [35] phenocopied the effects of Rab26 overexpression: it prevented glue leaving the salivary glands (Fig. 4n, p, compared with Fig. 4l). Interestingly, loss of Rab7 had no effect on glue release (Fig. 4m, p, compared with Fig. 4j). These data altogether indicate that Rab26 inhibits the acidification and maturation of glue granules, and Mon1 promotes Rab26 dissociation from these secretory vesicles that can be followed by Rab7 recruitment.

## Discussion

Since Rab26 was implicated in the regulation of secretory granule maturation and secretion in mammalian systems [20–22], we investigated the possible role of this small GTPase in the *Drosophila* salivary gland, an established model for the study of protein secretion. We found that Rab26 is present on small, immature glue-containing vesicles, and it dissociates from the larger granules, suggesting that it acts as a regulator of the early steps of the maturation process. Indeed, our loss-of-function studies revealed that glue granule maturation and acidification are accelerated in *rab26* mutants, and its overexpression delays this process. Acidification appears to be important for insulin granule secretion [15] and glue granule maturation in *Drosophila* [16]. Of note, Rab26 does not play a clear positive regulatory role in secretory granule maturation in fruit fly salivary gland cells [9]. In line with these, our data identify Rab26 as a negative regulator of secretory granule acidification and content reorganization in the Drosophila salivary gland.

Strikingly, knockout of Rab26 results in premature discharge, while its overexpression leads to retaining the glue in salivary glands, respectively (Fig. 4j–l). Interestingly, glue secretion from gland cells to the lumen was not blocked by Rab26 overexpression. Instead, glue could not leave the gland. This suggests that glue granule maturation is crucial for proper composition and viscosity of the mucinous content rather than for secretion itself. Of note, increased viscosity (thickening) of secretions prevents proper discharge from secretory glands, which is the major cause of cystic fibrosis symptoms [47].

Secretory granules can fuse with lysosomes to form degrading crinosomes where residual, non-secreted glue is recycled [12, 13]. Lysosomal fusions contribute to granule enlargement and eventually to uncontrolled secretion in the secretory glands of mice mutant for the Ca^2+^-permeable cation channel TRPML1 in a mucolipidosis type IV model [17], suggesting a connection between granule size and secretion. Our GFP-Lamp1 localization data show that Rab26 knockdown results in earlier fusions with lysosomes or Lamp carrier vesicles. Since secretory granules acidify during their maturation [15, 16], we propose that lysosomal transport plays a dual role in the progression of glue granules, being important for proper maturation and release as well as for the crinophagic degradation of residual granules that remain inside the cells after the wave of glue secretion.

Given the known role of factors responsible for glue granule–lysosome fusion in other types of lysosomal fusions [12, 14], we investigated the potential role of Rab26 in the maturation of autophagosomes and endosomes and the biogenesis of lysosome-related eye pigment granules. Since we did not see alterations in these pathways, our data suggest that Rab26 is a specific inhibitor of lysosomal transport to secretory granules.

During endosome maturation, the early endosomal small GTPase Rab5 is gradually replaced by Rab7, which enables the fusion of late endosomes with lysosomes [40]. We find that Rab26 is present on immature glue granules, and later it dissociates to allow the acquiring of lysosomal contents and acidification. We propose that a Rab26–Rab7 conversion mechanism may occur during secretory granule maturation, resembling the endosomal Rab5–Rab7 switch. Knockdown of Mon1, a subunit of the Mon1/Ccz1 guanine nucleotide exchange factor (GEF) complex that activates Rab7 [40], indeed inhibits dissociation of Rab26 from the glue granules, which thus enlarge and glue release is impaired. Interestingly, glue release was not affected by loss of Rab7, indicating that it is Mon1 that is somehow critical for removing Rab26 to allow further steps of secretory granule maturation.

We also investigated the localization of endogenous Rab7 upon loss of Rab26. Only a few structures are positive for Rab7 in controls, which are likely endosomes [7, 46] or secretory granules to be degraded [12]. Loss of type II phosphatidylinositol 4-kinase or retromer leads to defective glue granules, and their contents are transported to enlarged Rab7-positive vesicles [7, 46]. These data raise the possibility that Rab7 may be more important for degradation of defective, non-exocytosed, or excess granules than for their secretion. This is also consistent with the observation that Rab26 was shown to bind to RILP, a Rab7-interacting protein, on the surface of insulin granules to restrict them from secretion [23]. In our model system, many small Rab7-positive organelles form in *rab26* mutants before the wave of secretion, while there are bigger but fewer Rab7 vesicles after that. Indeed, residual non-secreted granules fuse with each other to form large organelles that will eventually be degraded [4] and glue granule degradation (crinophagy) requires Rab7 [12]. As Rab26 null mutation appears to accelerate glue granule maturation and secretion, the observed large Rab7 structures probably represent degrading crinosomes, the enlargement of which may be a result of the advanced stage of secretion and eventual crinosome formation.

Taken together, Rab26 seems to act as a positive regulator of glue granule maturation early on, possibly via promoting homotypic fusions of Golgi-derived glue-containing vesicles. Later, Rab26 leaves the surface of the larger granules in a Mon1-dependent manner to allow further steps of maturation before secretion, including lysosomal transport and acidification. Mature granules with proper mucin composition are eventually exocytosed and the remaining or defective ones are degraded by Rab7-dependent crinophagy (Fig. 8g). We have thus pinpointed Rab26 as a key regulator of secretory vesicle maturation, release, and degradation.

## Materials and methods

### Fly work

Flies were kept in glass tubes, on standard medium consisting of cornmeal, sucrose, and yeast. For the salivary gland experiments, late third-instar (wandering, or preparing for the puparium formation) larvae or prepupae were collected. Garland cells were dissected from late L3 stage (wandering) larvae. For starvation experiments, well-fed early L3 stage larvae were floated in 20% sucrose solution for 4 h.

The *UAS-YFP-Rab26* line (23245), the point mutant lines of *UAS-YFP-Rab26[Q250L]* (23243) and *UAS-YFP-Rab26[T204N]* (9807), the *w*^*1118*^ line (3605), the *EYFP-myc-Rab26* line (62555), the RNA interference line for Rab26 (JF31177) and the *sgs3-Sgs3-GFP* line (5884) were obtained from the Bloomington Drosophila Stock Center (BDSC), Bloomington, IN, USA. The RNA interference line for Mon1 (11926R-1) was obtained from the Fly Stocks of National Institute of Genetics (Nig-Fly), Mishima, Japan.

The null mutant *rab26[d1-2ex]* line was kindly provided by Robin Hiesinger, Division of Neurobiology, Institute for Biology, Freie Universität Berlin, Germany [28]. For analyzing lysosomal transport to glue granules in the salivary gland, we used the *UAS-GFP-Lamp1, sgs3-Sgs3-dsRed; fkh-Gal4* stock [12]. The *3xmCherry-Atg8a* transgene was generated by our group [35]. For generating clone cells expressing *UAS*-driven transgenes in the fat body, we used the *hs-Flp22;3xmCherry-Atg8a,UAS-GFP-Lamp1;Act* > *CD2* > *Gal4,UAS-Dcr2* stock [14]. For the garland cell experiments, we used the *pros-Gal4* driver [44]. For the eye experiments, the combined *w* + *;gmr-Gal4,ey-Gal4* stock was used, as previously [34, 43].

### Histology

For Lysotracker Red or Green (LTR, LTG) staining, we dissected larval salivary glands in phosphate-buffered saline (PBS), permeabilized them in 0.1% PBTX (0.1% Triton X-100 in PBS) containing also 0.05% sodium deoxycholate for 40 s (wandering larvae) or 20 s (larvae shortly before pupariation) (we omitted this step in case of prepupae), washed in PBS and incubated for 2 min in 100 nM LTR or LTG (Invitrogen) diluted in PBS. Then, we washed the specimens in PBS and transferred them to mounting solution (0.2 μg/ml 4′,6-diamidino-2-phenylindole (DAPI) as nuclear dye in 9:1 mixture of PBS and glycerin).

For immunostaining of larval salivary glands, we dissected them in PBS and permeabilized in 0.1% PBTX also containing 0.05% sodium deoxycholate for 20 s (larvae before puparium formation). In case of prepupae, we omitted this step. The specimens were fixed for 30 min in 4% paraformaldehyde (in PBS) at room temperature, then washed for 2 × 15 min in PBS, re-permeabilized in 0.1% PBTX for 10 min and incubated in 0.1% PBTX containing 10% bovine serum for 30 min. Then, the salivary glands were transferred to primary antibody solution diluted in 10% bovine serum-containing PBTX and incubated two days at 4 °C. The samples were then washed for 2 × 15 min in 0.1% PBTX, incubated in 0.1% PBTX containing 10% bovine serum for 30 min and transferred to the secondary antibody solution diluted in 10% bovine serum-containing PBTX for 4 h at room temperature. Then, the specimens were incubated in 0.1% PBTX containing Hoechst (1:200; as nuclear dye) (Sigma-Aldrich) and NaCl (4%), washed in 0.1% PBTX for 2 × 15 min and in PBS for 2 × 15 min. Specimens were mounted in Vectashield (Vector Laboratories). Immunostaining of garland nephrocytes of wandering third-instar larvae was performed as described previously [44].

For immunostainings, we used the primary antibodies monoclonal mouse anti-Rab7 (1:10, DSHB), polyclonal chicken anti-GFP (1:1000, Invitrogen) and polyclonal rabbit anti-Rab5 (1:100, Abcam, ab31261) and the secondary antibodies Alexa Fluor 568-conjugated anti-mouse and Alexa Fluor 488-conjugated anti-chicken and anti-rabbit (all 1:1000, Invitrogen).

### Western blot

Western blotting was performed as described before [48]. We used the primary antibodies polyclonal rabbit anti-Atg8a (1:3000, [48]) and monoclonal mouse anti-Tubulin (1:2000, DSHB) and the secondary antibodies alkaline phosphatase-conjugated anti-rabbit and anti-mouse (both 1:5000, Sigma-Aldrich).

### Electron microscopy

Dissected larval salivary glands were fixed in 3.2% paraformaldehyde, 1% glutaraldehyde (in case of samples for Gömöri’s acid phosphatase reaction, 2% paraformaldehyde, 2% glutaraldehyde), 1% sucrose and 0.003 M CaCl_2_ in 0.1 N sodium cacodylate buffer (pH 7.4), for overnight at 4 °C. Specimens were post-fixed in 0.5% osmium tetroxide for 1 h and in half-saturated aqueous uranyl acetate for 30 min, dehydrated in a graded series of ethanol, and embedded into TAAB 812 Resin Kit (T024) according to the manufacturer’s recommendations. A 70-nm section of the samples was stained in Reynold’s lead citrate, except those processed for Gömöri’s acid phosphatase reaction. Acid phosphatase cytochemistry was performed as described before [49].

Images were taken by a JEOL JEM-1011 transmission electron microscope equipped with a Morada camera (Olympus) and iTEM software (Olympus).

### Imaging and statistics

Fluorescent pictures were taken with an Axio Imager M2 microscope (Carl Zeiss) equipped with an Apotome2 grid confocal unit (Carl Zeiss), using EC Plan-Neofluar 40×/0.75 Air, Plan-Neofluar 2.5×/0.075 Air, Plan-Apochromat 40×/0.95 Air (salivary gland cells and fat cells), or Plan-Apochromat 63×/1.4 Oil (garland nephrocytes) objectives, an Orca Flash 4.0 LT sCMOS camera (Hamamatsu Photonics) and ZEN 2.3 software (Carl Zeiss). The compound eyes of adult animals were photographed on a Lumar V12 stereomicroscope equipped with an AxioCam ERc5s camera (Carl Zeiss).

Fluorescent structures (granule numbers and areas, endosome areas) were quantified manually or using ImageJ software (National Institutes of Health, Bethesda, MD, USA) by the same person. Cells were randomly selected for measurement of structures. In case of the mosaic system (Fig. S3c, d), the quantified control (GFP-negative) cells were immediate neighbors of the randomly selected, quantified knockdown (GFP-positive) cells. The threshold was set manually by the same person. For evaluating colocalization, we calculated Pearson’s coefficients by the Coloc 2 plugin of ImageJ (1, perfect colocalization; 0, no or incidental colocalization; − 1, mutually exclusive localization). The p values were calculated with nested two-tailed t tests (Figs. 1c, S1d, e, S4e) or nested one-way ANOVAs followed by Tukey’s multiple comparisons tests (Figs. 2h, i, 3d, 4o, 5j, 6e, 7g, 8e, f, S2a, e, f, S5f) as suggested by a recent article [50], using GraphPad Prism 9.4.1 for Windows software. To avoid confusion, in the figures we indicated only those p values that represent statistically significant and biologically relevant differences.

For producing the panels of the figures, fluorescent images were processed in ZEN 2.3 (Carl Zeiss) and Photoshop CS5 Extended (Adobe).

## Supplementary Information

Below is the link to the electronic supplementary material.

**Fig. S1: Additional Rab26 localization data. a:** YFP-Rab26 is found around the smaller Sgs3-dsRed secretory granules in the salivary gland of wandering stage larvae. **b:** YFP-Rab26[Q250L] remains localized on both smaller (green arrowheads) and larger granules (yellow arrowheads) at the end of the L3 stage. Moreover, the growth of secretory granules is further enhanced (compare Fig. 1a). **c:** RNA interference targeting Rab26 eliminates overexpressed YFP-Rab26[Q250L], demonstrating the efficiency of knockdown. Insets show merged images (top, M), Sgs3-dsRed channels (middle) and YFP-Rab26 or YFP-Rab26[Q250L] channels (bottom) with a 2x magnification enlarged from the boxed areas of the representative main panels (a-c). DAPI marks nuclei. **d-e:** Quantification of data shown in a (d) and b (e); n=50 randomly selected granules from 10 cells from 4 animals (d-e). Error bars denote SE and the number above the clasp shows p value (d). Sgs3-dsRed marks secretory granules. **f:** Punctate signal from EYFP knock in Rab26 is clearly visible compared to the adjacent control cells. Please note that salivary glands from control and EYFP-Rab26 larvae were imaged side-by-side on the same slide to facilitate visualization of faint YFP-Rab26 expression. Wand, wandering; bpf, before puparium formation. Scale bar, 20 µm (a-c, f). (TIF 13457 KB)

**Fig. S2: Additional glue granule size and acidification data. a: **Quantification of glue granule areas of control, *rab26* knockout and YFP-Rab26 overexpressing salivary glands at different stages; n=100 randomly selected granules from 10 cells from 4 animals. **b-d:** Glue granules are still not acidic in the salivary gland cells of wandering larvae (b), while Lysotracker Green shows extensive colocalization with the Sgs3-dsRed positive granules at later stages (c-d). Insets show merged images (top, M), Sgs3-dsRed channels (middle) and Lysotracker Green channels (bottom, LTG) with a 2x magnification enlarged from the boxed areas of the main panels (b-d). DAPI marks nuclei. **e:** Quantification of data shown in b-d; n=10 randomly selected cells from 4 animals. Box plot shows the data ranging between upper and lower quartiles; medians are indicated within the boxes. **f:** Quantification of data shown in Fig. 2a-g; n=10 cells from 4 animals. Error bars denote SE and the numbers above the clasps show p values. Wand, wandering; bpf, before puparium formation. Scale bar, 20 µm (b-d). (TIF 12874 KB)

**Fig. S3: Ultrastructure of glue secretion. a-c:** The ultrastructure of granular glue content changes to that of the secreted lumenal glue shortly before pupariation (a). Glue secretion to the lumen appears to take place normally in Rab26 mutant larvae (b). YFP-Rab26[Q250L] expression does not block secretion either (c). Bpf, before puparium formation. Scale bar, 1 µm (a-c). (TIF 6140 KB)

**Fig. S4:** **Rab26 status has no influence on autophagy. a:** Western blots of starved early 3^rd^ instar larval lysates do not show any significant changes in the levels of autophagosome-associated, lipidated (II) or non-lipidated (I) Atg8a forms in Rab26 mutants compared to controls. **b: **Western blots of starved early L3 stage larval lysates with Rab26 knockdown, or overexpressing GDP-locked versus GTP-locked point mutant forms of YFP-Rab26 using ubiquitous *daughterless*-Gal4 do not show any obvious changes in the levels of Atg8a forms compared to controls. **c-d:** Mosaic expression of GFP-Lamp1 (encircled with magenta) in 3xmCherry-Atg8a expressing fat tissue of starved early 3^rd^ instar larvae detects no difference in autophagosome-lysosome fusion between control (c) and Rab26 knockdown (d) fat cells. Magenta arrowheads show colocalizing structures. DAPI marks nuclei. **e:** Quantification of data shown in c-d; n=10 randomly selected cells from 4 animals. Box plot shows the data ranging between upper and lower quartiles; medians are indicated within the boxes. Error bars denote SE. Scale bar, 20 µm (c-d). (TIF 6526 KB)

**Fig. S5: Rab26 status has no effect on endocytic progression. a-e:** Rab5 positive early endosomes and Rab7 positive late endosomes are seen in a characteristic layered pattern in the garland cells of late 3^rd^ instar larvae (a). Neither Rab26 null mutation (b), nor Rab26 knockdown (c) leads to any alteration in the distribution or size of Rab5 and Rab7 organelles. Overexpression of a YFP-tagged wild type (d) or GTP-locked (e) form of Rab26 also results in normal Rab7 compartment and the YFP signal is diffuse throughout the cytosol. DAPI marks nuclei. **f:** Quantification of data shown in a-e; n=287 (a), n=322 (b), n=231 (c), n=232 (d), n=219 (e) endosomes from 10 cells from 4 animals. Error bars denote SD. Wand, wandering. Scale bar, 10 µm (a-e). (TIF 17708 KB)

**Fig. S6: Rab26 status does not influence eye color. a-d:** Normal compound eye pigmentation and morphology is seen upon eye-specific expression of a Rab26 RNA interference construct (b), wild type (c) or Q250L point mutant (d) forms of YFP-Rab26, similarly to the control flies (a). (TIF 3369 KB)

## Data Availability

Source data including original unmodified images and statistics are available from the authors on request.
