# Supplementary figures and images for: Rab26 controls secretory granule maturation and breakdown in Drosophila
Source: Cell Mol Life Sci. 2023 Jan 4;80(1):24. doi: 10.1007/s00018-022-04674-8 (PMC9813115; doi:10.1007/s00018-022-04674-8)

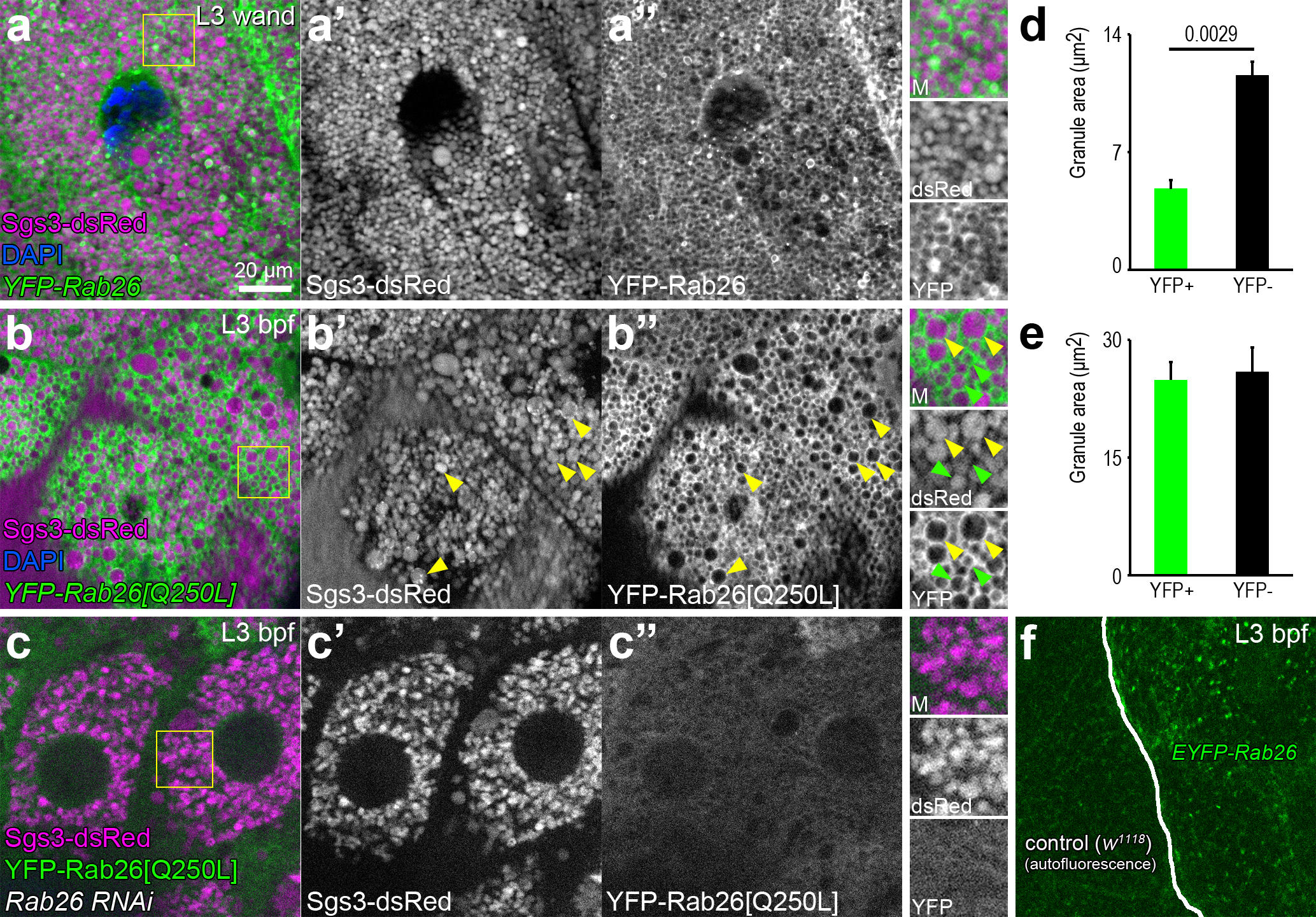

Supplement: Supplementary file 1 — Fig. S1: Additional Rab26 localization data. a: YFP-Rab26 is found around the smaller Sgs3-dsRed secretory granules in the salivary gland of wandering stage larvae. b: YFP-Rab26[Q250L] remains localized on both smaller (green arrowheads) and larger granules (yellow arrowheads) at the end of the L3 stage. Moreover, the growth of secretory granules is further enhanced (compare Fig. 1a). c: RNA interference targeting Rab26 eliminates overexpressed YFP-Rab26[Q250L], demonstrating the efficiency of knockdown. Insets show merged images (top, M), Sgs3-dsRed channels (middle) and YFP-Rab26 or YFP-Rab26[Q250L] channels (bottom) with a 2x magnification enlarged from the boxed areas of the representative main panels (a-c). DAPI marks nuclei. d-e: Quantification of data shown in a (d) and b (e); n=50 randomly selected granules from 10 cells from 4 animals (d-e). Error bars denote SE and the number above the clasp shows p value (d). Sgs3-dsRed marks secretory granules. f: Punctate signal from EYFP knock in Rab26 is clearly visible compared to the adjacent control cells. Please note that salivary glands from control and EYFP-Rab26 larvae were imaged side-by-side on the same slide to facilitate visualization of faint YFP-Rab26 expression. Wand, wandering; bpf, before puparium formation. Scale bar, 20 µm (a-c, f). (TIF 13457 KB) [file 18_2022_4674_MOESM1_ESM.tif]

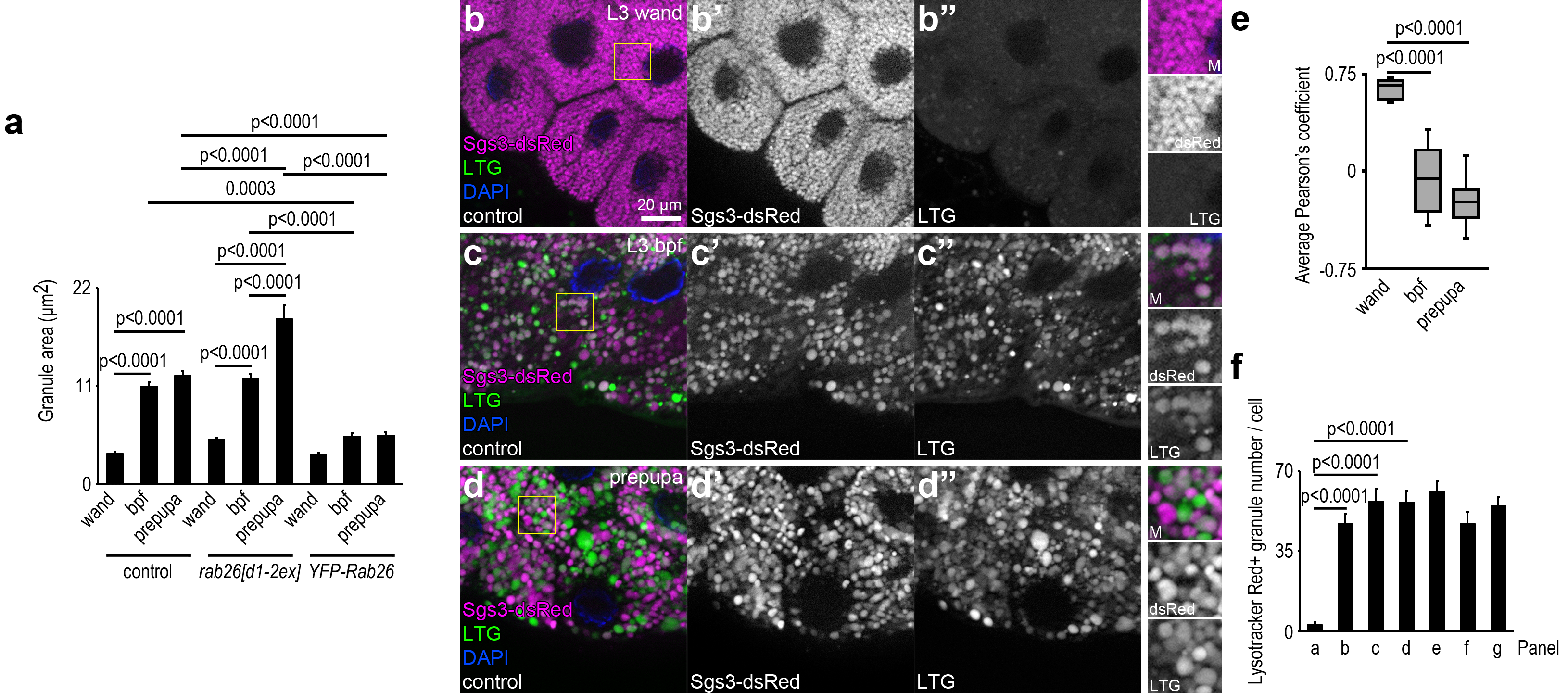

Supplement: Supplementary file 2 — Fig. S2: Additional glue granule size and acidification data. a: Quantification of glue granule areas of control, rab26 knockout and YFP-Rab26 overexpressing salivary glands at different stages; n=100 randomly selected granules from 10 cells from 4 animals. b-d: Glue granules are still not acidic in the salivary gland cells of wandering larvae (b), while Lysotracker Green shows extensive colocalization with the Sgs3-dsRed positive granules at later stages (c-d). Insets show merged images (top, M), Sgs3-dsRed channels (middle) and Lysotracker Green channels (bottom, LTG) with a 2x magnification enlarged from the boxed areas of the main panels (b-d). DAPI marks nuclei. e: Quantification of data shown in b-d; n=10 randomly selected cells from 4 animals. Box plot shows the data ranging between upper and lower quartiles; medians are indicated within the boxes. f: Quantification of data shown in Fig. 2a-g; n=10 cells from 4 animals. Error bars denote SE and the numbers above the clasps show p values. Wand, wandering; bpf, before puparium formation. Scale bar, 20 µm (b-d). (TIF 12874 KB) [file 18_2022_4674_MOESM2_ESM.tif]

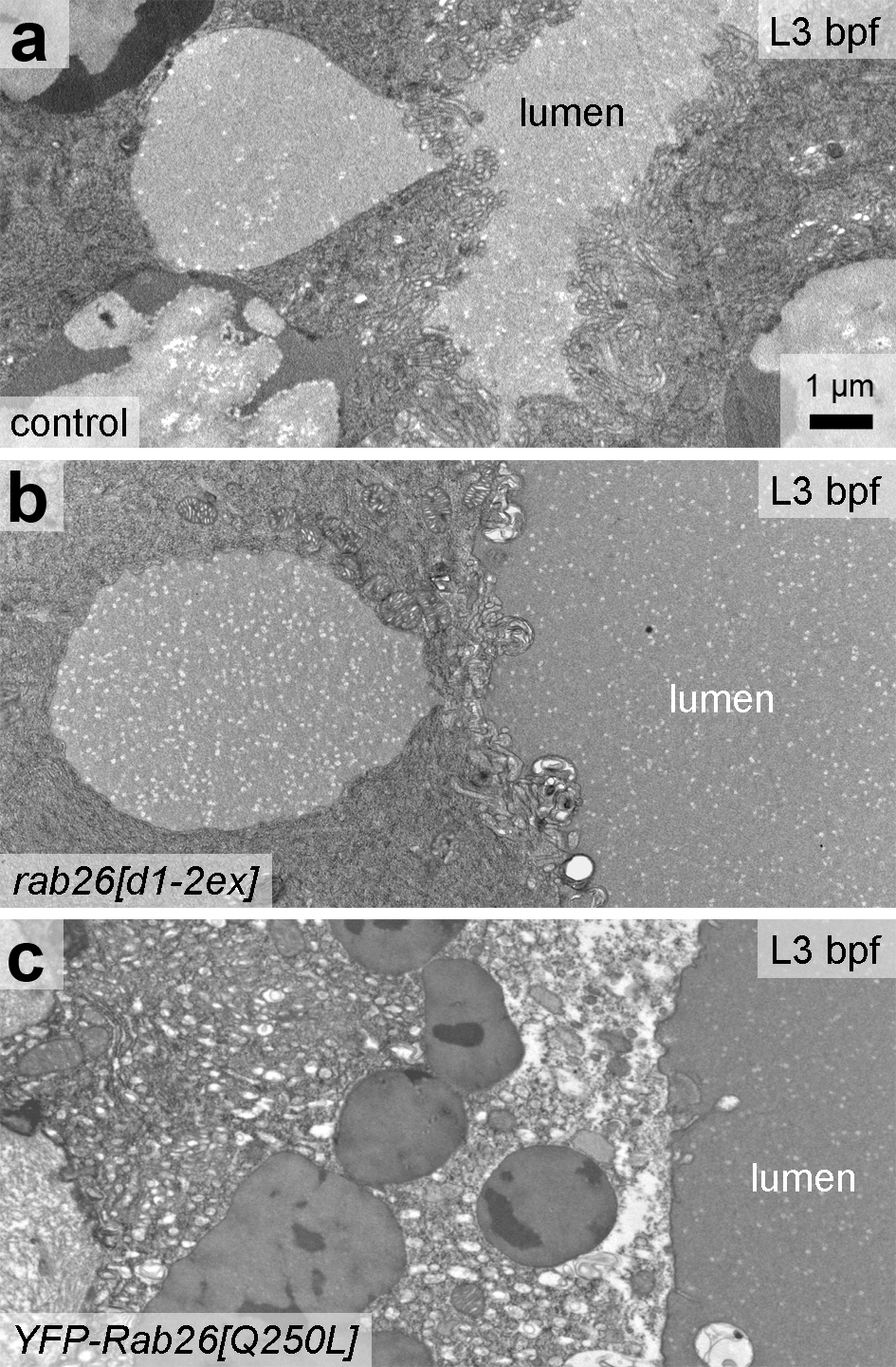

Supplement: Supplementary file 3 — Fig. S3: Ultrastructure of glue secretion. a-c: The ultrastructure of granular glue content changes to that of the secreted lumenal glue shortly before pupariation (a). Glue secretion to the lumen appears to take place normally in Rab26 mutant larvae (b). YFP-Rab26[Q250L] expression does not block secretion either (c). Bpf, before puparium formation. Scale bar, 1 µm (a-c). (TIF 6140 KB) [file 18_2022_4674_MOESM3_ESM.tif]

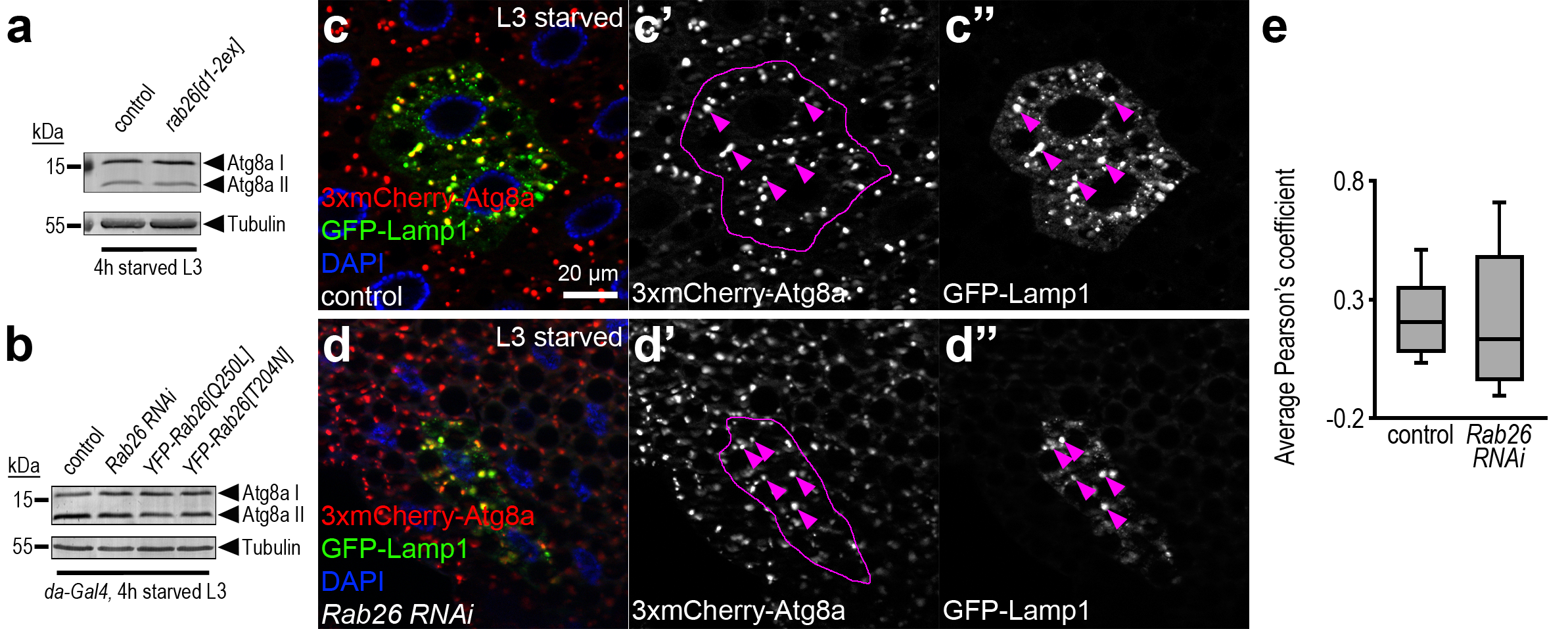

Supplement: Supplementary file 4 — Fig. S4: Rab26 status has no influence on autophagy. a: Western blots of starved early 3rd instar larval lysates do not show any significant changes in the levels of autophagosome-associated, lipidated (II) or non-lipidated (I) Atg8a forms in Rab26 mutants compared to controls. b: Western blots of starved early L3 stage larval lysates with Rab26 knockdown, or overexpressing GDP-locked versus GTP-locked point mutant forms of YFP-Rab26 using ubiquitous daughterless-Gal4 do not show any obvious changes in the levels of Atg8a forms compared to controls. c-d: Mosaic expression of GFP-Lamp1 (encircled with magenta) in 3xmCherry-Atg8a expressing fat tissue of starved early 3rd instar larvae detects no difference in autophagosome-lysosome fusion between control (c) and Rab26 knockdown (d) fat cells. Magenta arrowheads show colocalizing structures. DAPI marks nuclei. e: Quantification of data shown in c-d; n=10 randomly selected cells from 4 animals. Box plot shows the data ranging between upper and lower quartiles; medians are indicated within the boxes. Error bars denote SE. Scale bar, 20 µm (c-d). (TIF 6526 KB) [file 18_2022_4674_MOESM4_ESM.tif]

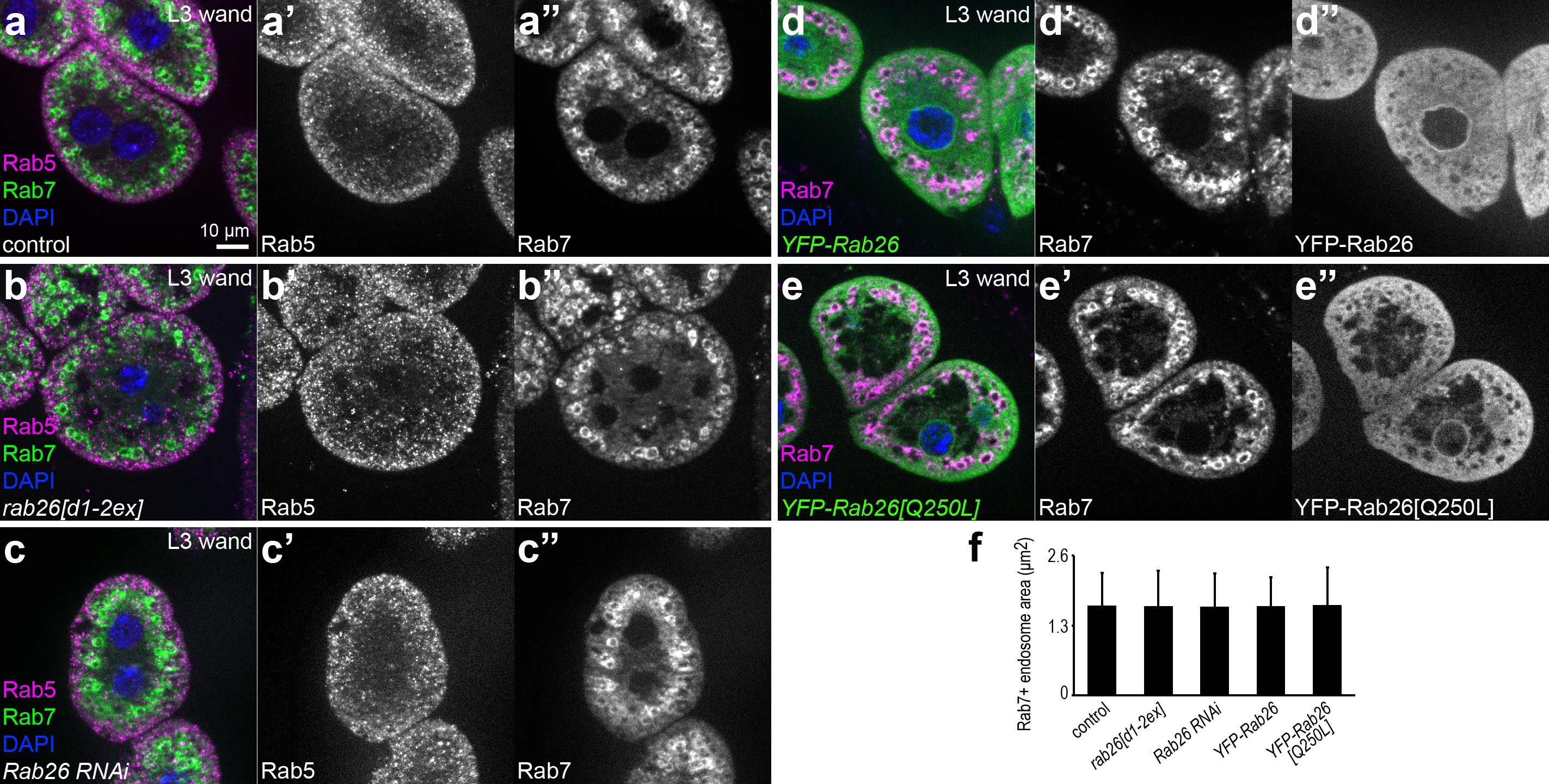

Supplement: Supplementary file 5 — Fig. S5: Rab26 status has no effect on endocytic progression. a-e: Rab5 positive early endosomes and Rab7 positive late endosomes are seen in a characteristic layered pattern in the garland cells of late 3rd instar larvae (a). Neither Rab26 null mutation (b), nor Rab26 knockdown (c) leads to any alteration in the distribution or size of Rab5 and Rab7 organelles. Overexpression of a YFP-tagged wild type (d) or GTP-locked (e) form of Rab26 also results in normal Rab7 compartment and the YFP signal is diffuse throughout the cytosol. DAPI marks nuclei. f: Quantification of data shown in a-e; n=287 (a), n=322 (b), n=231 (c), n=232 (d), n=219 (e) endosomes from 10 cells from 4 animals. Error bars denote SD. Wand, wandering. Scale bar, 10 µm (a-e). (TIF 17708 KB) [file 18_2022_4674_MOESM5_ESM.tif]

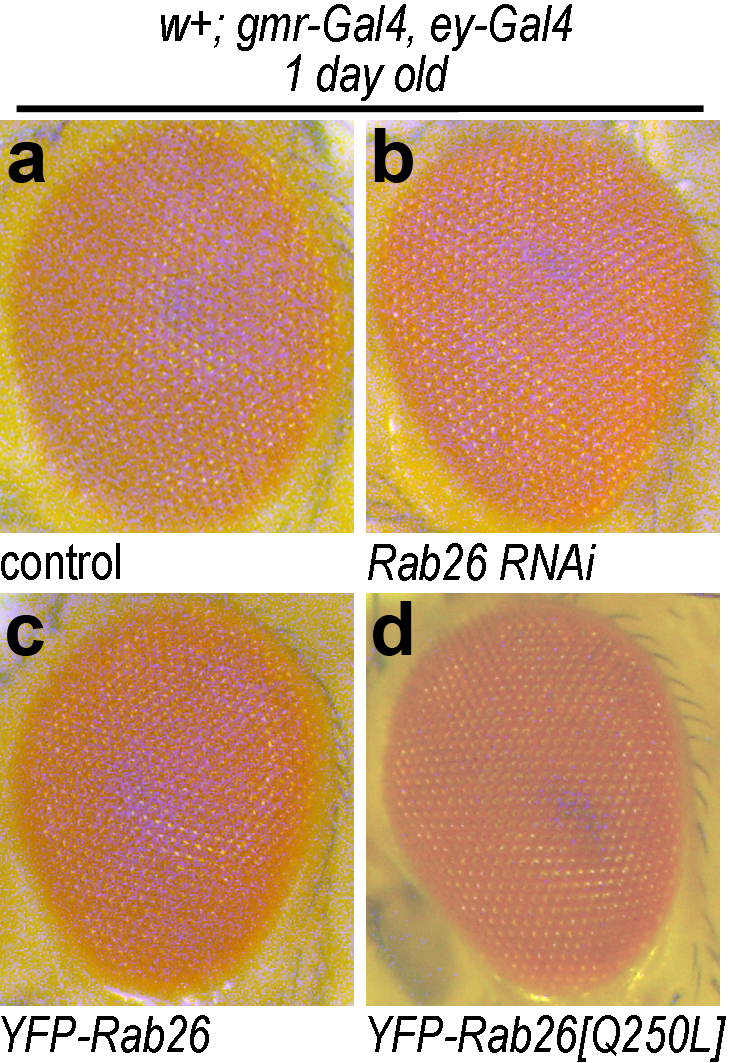

Supplement: Supplementary file 6 — Fig. S6: Rab26 status does not influence eye color. a-d: Normal compound eye pigmentation and morphology is seen upon eye-specific expression of a Rab26 RNA interference construct (b), wild type (c) or Q250L point mutant (d) forms of YFP-Rab26, similarly to the control flies (a). (TIF 3369 KB) [file 18_2022_4674_MOESM6_ESM.tif]
